# Worcester Hospitals' Monthly Collections Scheme

**Published:** 1913-08-02

**Authors:** 


### Worcester Hospitals' Monthly Collections Scheme.

On the 21st ult. the collectors for this organisation were most hospitably entertained by Mr. and Mrs. Ferguson Chance at Blackmore Park. The company much enjoyed the beautiful grounds, and at 4 p.m. assembled for tea, which was served in a marquee. After tea a cordial vote of thanks to their host and hostess was proposed by Mr. Shrewsbury Smith, chairman of the committee of the Worcester General Infirmary, and seconded by the Rev. F. W. Davenport, representing the Malvern Hospital, which also profits by the monthly collections. Mr. Ferguson Chance, in returning thanks, expressed the pleasure he and Mrs. Chance had in welcoming those who were engaged in such good work for the hospitals. Both hospitals represented—the Worcester General Infirmary, about 170 years old, and the Malvern Rural Hospital, a comparatively new institution—were of great importance, and were doing excellent work. One thing was certain, that whatever the result of the Insurance Act (not altogether a bad Act) might be, hospitals would be needed quite as much as ever. Panel doctors could not attend to the very severe cases, which would of necessity come to the infirmaries, where they would get the best appliances, the best treatment, and the best nurses. Alderman Leicester, in moving a hearty vote of thanks to the ladies who had collected for that fund, made an earnest appeal on behalf of the Worcester Infirmary. He said it would be an awful reflection upon the people of the early part of the twentieth century if ever the institution should cease to exist. It was built by their forefathers, who had also to pay the cost of running it during their lifetime, and set aside much money to endow it. They of the present day were not asked to build or to find funds for endowment; they were only asked to do a third of what their forefathers did—namely, to provide the necessary funds to run it for the relief of the sick and suffering poor of the county.

